# Quality-Related Process Monitoring and Diagnosis of Hot-Rolled Strip Based on Weighted Statistical Feature KPLS

**DOI:** 10.3390/s23136038

**Published:** 2023-06-29

**Authors:** Hesong Guo, Jianliang Sun, Junhui Yang, Yan Peng

**Affiliations:** 1 School of Mechanical Engineering, Yanshan University, Qinhuangdao 066004, China; guohesong@stumail.ysu.edu.cn (H.G.); 17884809331@163.com (J.Y.); pengyan@ysu.edu.cn (Y.P.); 2 National Engineering Research Center for Equipment and Technology of Cold Rolled Strip, Yanshan University, Qinhuangdao 066004, China

**Keywords:** data weighting, quality-related, fault detection, hot rolled strip, KPLS

## Abstract

Rolling is the main process in steel production. There are some problems in the rolling process, such as insufficient ability of abnormal detection and evaluation, low accuracy of process monitoring, and fault diagnosis. To improve the accuracy of quality-related fault diagnosis, this paper proposes a quality-related process monitoring and diagnosis method for hot-rolled strip based on weighted statistical feature KPLS. Firstly, the process-monitoring and diagnosis model of strip thickness and quality based on the KPLS method is introduced. Then, considering that the KPLS diagnosis method ignores the contribution of process variables to quality, it is easy to misjudge the root cause of quality in the diagnosis process. Based on the rolling mechanism model, the influence weight of strip thickness is constructed. By weighing the statistical data features, a quality diagnosis framework of series structure data fusion is constructed. Finally, the method is applied to the 1580 mm hot-rolling process for industrial verification. The verification results show that the proposed method has higher diagnostic accuracy than PLS, KPLS, and other methods. The results show that the diagnostic model based on weighted statistical feature KPLS has a diagnostic accuracy of more than 96% for strip thickness and quality-related faults.

## 1. Introduction

Quality-related process monitoring and diagnosis are crucial for maintaining a high level of industrial development and product upgrading [1,2]. To ensure production safety and improve product quality, the comprehensive intelligent development of manufacturing industry puts forward new requirements for product quality inspection of process industry [3,4,5]. In the process of hot rolling, there are many process parameters related to the final product quality, such as width and thickness, and the relationship between quality factors and quality indicators is unclear. How to quickly monitor and diagnose the abnormal quality fluctuations in the rolling process, locate the causes of abnormalities, and improve the product quality has become an important problem in the production process of strip [6]. In recent years, to improve the production efficiency in industrial processes, industrial production equipment and processes have become more complex. Once the process abnormality or fault transfer occurs, it is very likely to cause a series of cascading failures leading to serious production accidents, and even cause serious economic losses and casualties. Therefore, how to carry out timely and accurate anomaly detection of the production processes to ensure its production safety and product quality has become an urgent problem to be solved in industry and academia [7,8].

For the monitoring and diagnosis of product quality in the production process, a lot of research work has been carried out. Based on multivariate statistical process control (MSPC), the quality-related process monitoring the multivariate production process is realized, including canonical correlation analysis (CCA), principal component analysis (PCA), principal component regression (PCR), partial least squares (PLS), etc. [9,10,11,12]. Although the above methods can effectively monitor the fluctuations and deviations in process variables, these contributions focus on finding whether there are abnormal fluctuations, ignoring the contribution of abnormal process parameters to quality indicators and the impact of nonlinearity on monitoring results. Therefore, it is necessary to study the influence of nonlinearity on quality diagnosis and the root causes of product quality defects.

Kernel mapping is used to solve nonlinear problems in the monitoring and diagnosis process [13,14]. Considering the autocorrelation between process data, Wang XM [15] carried out CVA in the kernel space generated by kernel principal component analysis (KPCA), and established the kernel CVA (KCVA) model to monitor the nonlinear dynamic process, which significantly improved the fault detection performance. To detect the early faults of nonlinear industrial processes, a probability-dependent nonlinear statistical monitoring framework is constructed based on KPCA, which measures the probability distribution changes caused by small displacements [16]. Qi L [17] embedded singular value decomposition (SVD) into kernel principal component regression (KPCR) to achieve quality-related process monitoring at a lower computational cost. The KPLS method can obtain the features of the tested object during the detection process, so it has been widely applied in quality-related fault detection [18]. Jiao J [19] proposed a nonlinear quality-related fault detection method based on kernel partial least squares (KPLS) model to deal with the nonlinear characteristics between variables. QL Jia [20] proposed a new dynamic kernel least squares (D-KPLS) modeling method and the corresponding process monitoring method, established a more robust input-output variable relationship than the standard KPLS model, and realized the quality-related process monitoring and prediction. Fezai R [21] et al. established two different interval-reduced KPLS (IRKPLS) models for monitoring large-scale nonlinear uncertain systems. Kong [22] proposes an efficient KPLS model and introduces an orthogonal signal correction (OSC) preprocessing method based on quality estimation. The OSC preprocessing is performed on the measured data through quality estimation values, reducing computational complexity and false alarm rate. To solve the problem that KPLS can only extract shallow features from process measurements, a hierarchical feature learned by Stacked Sparse Automatic Encoder (SSAE) was used as input to establish an SSAE KPLS model for process monitoring based on nonlinear relationships between variables [23]. Liang Ma [24] proposed a method combining adaptive kernel rule variable analysis and Bayesian fusion for real-time, hierarchical detection and quality-related multi-fault detection. This method adapts to increasingly complex industrial process and reduces the time complexity brought by the recursive process. Jiao JF [25] integrated the idea of kernel sample equivalent substitution (KSER) into the KPLS model, established a linear regression between the original sample and the output, and solved the complex nonlinear process diagnosis problem with extremely computational complexity. In addition, some scholars used the neural network [26,27], k-Nearest Neighbor algorithm [28], and other intelligent algorithms to identify and diagnose the quality-related faults [29].

Although the above methods can identify the causes of abnormal features in process monitoring and diagnosis, it is difficult to effectively distinguish the degree of influence of process variables on quality under complex multivariate conditions. At the same time, the nonlinear mapping of the kernel method completely covers up the correspondence between the original variable data samples and the model. In response to the above issues, traditional methods mainly improve the diagnostic accuracy of quality defects by improving algorithms or data quality, ignoring the impact of various variables on quality, and lacking the mechanical explanation of process variables on quality. Thus, the quality diagnosis process misjudges the source of the abnormality. Therefore, in this paper, the mechanism model is selected to establish the influence weight of strip thickness, and the improved statistical feature data set is obtained by weighted statistical features, and propose the quality-related process monitoring and diagnosis of hot-rolled strip based on weighted statistical feature KPLS. Compared with the traditional quality-related process monitoring and diagnosis algorithm, the KPLS algorithm based on weighted statistical characteristics improves the physical interpretation of process variables on strip quality. Considering the degree of influence of different variables on quality, the weight of mass variable influence is constructed based on rolling mechanism model. Improve the detection rate of quality-related failures by influencing weight reconstruction monitoring model input data. In addition, the data model is improved in the form of data weighting, which can solve the problem that the fault characteristics of industrial processes are not significant due to small amplitude or slow change. Finally, the industrial verification of the model is carried out by using the production data of the 1580 hot rolling production line. The results meet the requirements of the field quality detection and can effectively identify the abnormal fluctuation variables.

The remaining sections are arranged as follows: Section 2 introduced the monitoring and diagnosis model of the KPLS algorithm for strip quality. In Section 3, the weight of thickness quality influence was constructed based on the rolling process, and the data set was weighted and reconstructed by the weight. A series structure model of quality-related fault diagnosis based on mechanism data fusion was established. The verification experiment and summary are found in Section 4 and Section 5, respectively.

## 2. Monitoring and Diagnosis of Quality-Related Faults of Hot-Rolled Strip Based on KPLS

A large amount of data generated in the production process of hot rolled strip have strong nonlinearity. Aiming at the nonlinearity of the plate and strip rolling process. Given input matrix X consisting of n samples with m process variables, and output matrix Y containing n observations with p quality variables, respectively, i.e.,

$$X=\left[\begin{array}{c} x_{1}\\ x_{2}\\ \begin{array}{c} \vdots \\ x_{n} \end{array} \end{array}\right]\in R^{n\times m},\;Y=\left[\begin{array}{c} y_{1}\\ y_{2}\\ \begin{array}{c} \vdots \\ y_{n} \end{array} \end{array}\right]\in R^{n\times p},$$

where $x_{i}\in R^{m}$ and $y_{i}\in R^{p}\;(i=1, 2,\cdots ,n)$ are row vectors, respectively.

Using the nonlinear mapping function, it is a common way [20] to map the original input data to a high-dimensional feature space. To this aim, given a nonlinear projection function ϕ, has ϕ:x→ϕ(x). The data mapping matrix of high dimensional space is obtained Φ.

(1) $$\Phi ={\left[\phi \left(x_{1}\right),\phi \left(x_{2}\right),...,\phi \left(x_{n}\right)\right]}^{T}\in R^{n}$$

The element $K_{ij}$ of the *i*th row and *j*th column of kernel matrix K is

(2) $$K_{ij}=K\left(x_{i},x_{j}\right)={\phi \left(x_{i}\right)}^{T}\phi \left(x_{j}\right)=\langle \phi \left(x_{i}\right),\phi \left(x_{j}\right)\rangle$$

Due to the diversity of mapping functions, including polynomial, sigmoid, and other kernel functions, these kernel functions introduce multiple parameters. However, the Gaussian kernel function only has one parameter σ, which is beneficial for controlling model accuracy. Therefore, this article selects Gaussian kernel function to calculate the kernel matrix.

(3) $$K\left(x_{i},x_{j}\right)=exp\left(-\frac{{\left\|x_{i}-x_{j}\right\|}^{2}}{2\sigma^{2}}\right)$$

where $K\left(x_{i},x_{j}\right)$ is Gaussian kernel function and σ is constant, σ=16, i=1,2,⋯,n, j=1,2,⋯,n, respectively.

The following kernel matrix K is usually defined [30] to avoid the explicit using of Φ:

(4) $$K=\Phi \Phi^{T}$$

Centralize the kernel matrix.

(5) $$\bar{K}=K-\frac{1}{n}I_{n}K-\frac{1}{n}KI_{n}+\frac{1}{n^{2}}I_{n}KI_{n}$$

(6) $$I_{n}=\left[\begin{array}{ccc} 1 & \cdots & 1\\ \vdots & \ddots & \vdots \\ 1 & \cdots & 1 \end{array}\right]\in R^{n\times n}$$

The kernel matrix of the sample to be monitored is constructed, and the new data in the kernel matrix of the feature space is centralized by using the training data.

(7) $$K_{new}=\left[k\left(x_{1},x_{new}\right),k\left(x_{2},x_{new}\right),\cdots ,k\left(x_{n},x_{new}\right)\right]$$

(8) $${\bar{K}}_{new}=K_{new}-\frac{1}{n}I_{N}\bar{K}-\frac{1}{n}K_{new}I_{n}+\frac{1}{n^{2}}I_{N}\bar{K}I_{n}$$

(9) $$I_{N}=\left[1\cdots 1\right]\in R^{1\times n}$$

Construct KPLS [31] model.

(10) $$\left\{\begin{array}{c} K=\Phi +\Phi_{r}=TP^{T}+K_{r}\\ Y=Y+Y_{r}=UQ^{T}+Y_{r} \end{array}\right.$$

(11) $$T=\bar{\Phi }R$$

(12) $$R={\bar{\Phi }}^{T}U{\left(T^{T}\bar{K}U\right)}^{-1}$$

The above formulas P and Q are the load matrix of K and Y respectively, T and U are the score matrix of K and Y, respectively.

Sample score matrix to be tested.

(13) $$t_{new}={\bar{K}}_{new}U{\left(T^{T}\bar{K}U\right)}^{-1}$$

Build monitoring statistics [22].

(14) $$\left\{\begin{array}{l} T^{2}=t_{\mathrm{new}}^{T}\Lambda^{-1}t_{\mathrm{new}}\\ \mathrm{SPE}={\left\|{\tilde{x}}_{\mathrm{new}}\right\|}^{2} \end{array}\right.$$

The covariance matrix of the training sample score is $\Lambda =\frac{T^{T}T}{n-1}$.

The $T^{2}$ statistic follows the F distribution, and the SPE statistic is based on the assumption of standard normal distribution. The control limits are calculated as follows.

(15) $$\left\{\begin{array}{l} T_{\mathrm{ucl}}^{2}\left(\alpha_{0}\right)=\frac{A\left(n^{2}\;-\;1\right)}{n\left(n-A\right)}F_{A,n\;-\;A,\alpha_{0}}\\ {\mathrm{SPE}}_{\mathrm{ucl}}^{2}\left(\alpha_{0}\right)=g\chi_{h,\alpha_{0}}^{2} \end{array}\right.$$

where $g=\frac{S}{2\mu }$, $h=\frac{2\mu^{2}}{S}$, μ is the average value of SPE statistics, S is the variance, $\alpha_{0}$ is the significance level, A is the number of principal elements determined by cross validation, respectively.

The nonlinear contribution plot is used to calculate the contribution of the parameters in the sample points to the monitoring indicators, find out the fault variables that affect the quality, and realize the identification and diagnosis of abnormal causes in the production process.

For the new test sample $x_{new,l}\in R^{1\times m}\left(l=1,2,\cdots ,m\right)$ of the new input point, the partial derivatives of the nuclear matrix $K_{new}$ of the new input point for each parameter are as follows.

(16) $${\bar{K}}_{new,i}^{\prime }=K_{new,i}^{\prime }-K_{new,i}^{\prime }I_{n}$$

Calculate the SPE contribution value of each parameter.

(17) $$cont=\left|{cont}^{*}\right|$$

(18) $${cont}^{*}=-\frac{2}{n}\sum_{i=1}^{n}{\bar{K}}_{new,i}^{\prime }-K_{new}^{\prime }(2Tt_{new}^{T}-UT^{T}\bar{K}Tt_{new}^{T})+t_{new}T^{T}\bar{K}TU^{T}{\bar{K}}^{\prime }{}_{new}^{T}$$

where ${\bar{K}}_{new}^{\prime }$ is the partial derivative vector ${\bar{K}}_{new,i}^{\prime }$, which forms the partial derivative kernel matrix.

The monitoring and diagnosis of quality-related faults of hot-rolled strip based on KPLS is solved by Algorithm 1.
**Algorithm 1:** The calculation algorithm of KPLS [19]Step 1Set i=1, $Y_{1}=Y$, ${\bar{K}}_{1}=\bar{K}$
Step 2Select the first column of $Y_{i}$ as $u_{i}$.Step 3$t_{i}={\bar{K}}_{i}u_{i}$Step 4$t_{i}=\frac{t_{i}}{\left\|t_{i}\right\|}$Step 5$c_{i}=Y_{i}^{T}t_{i}$, $u_{i}=Y_{i}t_{i}$Step 6$u_{i}=\frac{u_{i}}{\left\|u_{i}\right\|}$Step 7Repeat step 3 to step 6 until $t_{i}$ converges.Step 8${\bar{K}}_{i+1}=\left(I_{N}-t_{i}t_{i}^{T}\right){\bar{K}}_{i}\left(I_{N}-t_{i}t_{i}^{T}\right)$; $Y_{i+1}=\left(I_{N}-t_{i}t_{i}^{T}\right)Y_{i}$Step 9Get parameters $T=\left[T,t_{i}\right]$, $U=\left[U,u_{i}\right]$
Step 10Set i=i+1 and repeat step 2 to step 9 until i>r



## 3. KPLS Quality-Related Process Monitoring and Diagnosis Based on Data Weighting

In the above KPLS monitoring model, the monitoring and diagnosis of quality-related faults of hot rolled plates and strips can be realized, but there is a lack of reasonable physical explanation for each variable, resulting in a misjudgment of the cause of quality defects. Thus, a method for quality-related process monitoring and diagnosis of hot-rolled strips based on the weighted statistical feature KPLS (WKPLS) was proposed.

### 3.1. Introduction to the Hot Rolling Process

**Figure 1:**
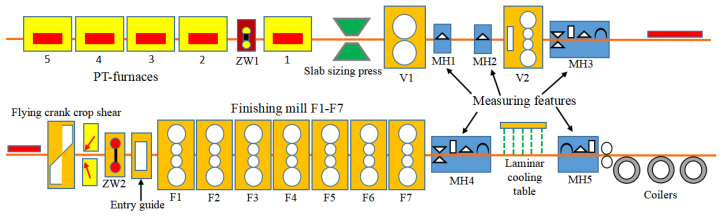
Multi-process collaborative strip production.

Hot rolling is a complex multi-process collaborative industrial production process [32]. It mainly includes heating, roughing mill, finishing mill, laminar cooling, coiling, and other processes, as shown in Figure 1. The finishing mill stage is usually composed of 5–7 stands. Each stand of finishing mill is mainly composed of frame, work roll, back-up roll, hydraulic screw down, bending roll device, corresponding hydraulic control system, feedback control, and signal data acquisition system.

The strip is affected by rolling force and other related parameters. The metal produces elastic–plastic deformation between the roll gap, and the abnormal quality fluctuation of the strip is caused by the abnormal rolling parameters [33]. The thickness difference of strip is affected by the abnormal fluctuation of parameters such as inlet thickness change, temperature change, tension change, rolling speed change, and roll gap change. To improve quality-related process monitoring and diagnostic accuracy. In this paper, the influence coefficient of thickness in rolling process is analyzed by the thickness difference equation of the steady state rolling process. Based on the influence coefficient of thickness difference, the index weight of thickness influence coefficient is established. This improves the data model’s ability to physically interpret process variables.

### 3.2. Strip Thickness Influence Weight

The rolling spring curve of strip steel is the basis of this study, and the thickness depends on the three main factors of no-load roll gap, rolling mill modulus, and rolling force [34]. According to the bounce curve, the outlet thickness h can be expressed by the following formula.

(19) $$h=S_{0}^{\prime }+\frac{P-P_{0}}{M}-\left(\delta -\delta_{0}\right)$$

where $S_{0}^{\prime }$ is the actual roll gap, P and $P_{0}$ refer to the rolling force and preloading force, respectively. δ and $\delta_{0}$ are the oil film thickness of liquid friction bearing and the oil film thickness at the artificial zero position, respectively. M is the longitudinal stiffness modulus of the rolling mill.

Stiffness of rolling mill is the definite value of rolling mill. The gap position between the rollers remains unchanged, and the thickness of the strip outlet is affected by the change in the rolling force. According to the bouncing equation, the thickness increment takes the following form.

(20) $$∆h=∆S_{0}+\frac{∆P}{M}-∆\delta$$

The expression of longitudinal thickness difference can be changed to the following formula. Note that ∆h is calculated in detail as shown in Appendix A.

(21) $$∆h=\left(∆S_{0}+\frac{∆F}{M}\right)/\left(1+\frac{W}{M}\right)$$

(22) $$∆F=\frac{\partial P}{\partial H}\cdot ∆H+\frac{\partial P}{\partial T_{b}}\cdot ∆T_{b}+\frac{\partial P}{\partial T_{f}}\cdot ∆T_{f}+\frac{\partial P}{\partial \mu }\cdot ∆\mu +\frac{\partial P}{\partial K}\cdot ∆K$$

Based on the thickness increment equation of the rolling process, the influence coefficients of the thickness difference ∆H, the back tension increment $∆T_{b}$, the front tension increment $∆T_{f}$, the friction coefficient ∆μ, the deformation resistance ∆K and other parameters on the thickness difference ∆h can be obtained. As shown below.

(23) $$Q_{H}=\frac{∆h}{∆H}=\frac{\partial P}{\partial H}\cdot \frac{1}{M+W}$$

(24) $$Q_{T_{b}}=\frac{∆h}{∆T_{b}}=\frac{\partial P}{\partial T_{b}}\cdot \frac{1}{M+W}$$

(25) $$Q_{T_{f}}=\frac{∆h}{∆T_{f}}=\frac{\partial P}{\partial T_{f}}\cdot \frac{1}{M+W}$$

(26) $$Q_{\mu }=\frac{∆h}{∆\mu }=\frac{\partial P}{\partial \mu }\cdot \frac{1}{M+W}$$

(27) $$Q_{K}=\frac{∆h}{∆K}=\frac{\partial P}{\partial K}\cdot \frac{1}{M+W}$$

In the multi-stand rolling process, each stand controls the longitudinal thickness differently. The equivalent longitudinal stiffness of the rolling mill can be obtained.

(28) $$M_{\alpha }=\frac{M}{1-\alpha }$$

where, α is the compensation coefficient.

F1 and F2 stands set a larger equivalent stiffness modulus so that α=0.2. F3 stand is natural stiffness, taking α=0. F4–F7 stand adopts a small compensation coefficient and obtains better flatness quality through soft characteristic control, in this paper α=−0.2.

Order $\varphi =\frac{1}{M_{\alpha }+W}$, The influence coefficient of each variable on the thickness difference can be obtained, as shown in Table 1.

**Figure 2:**
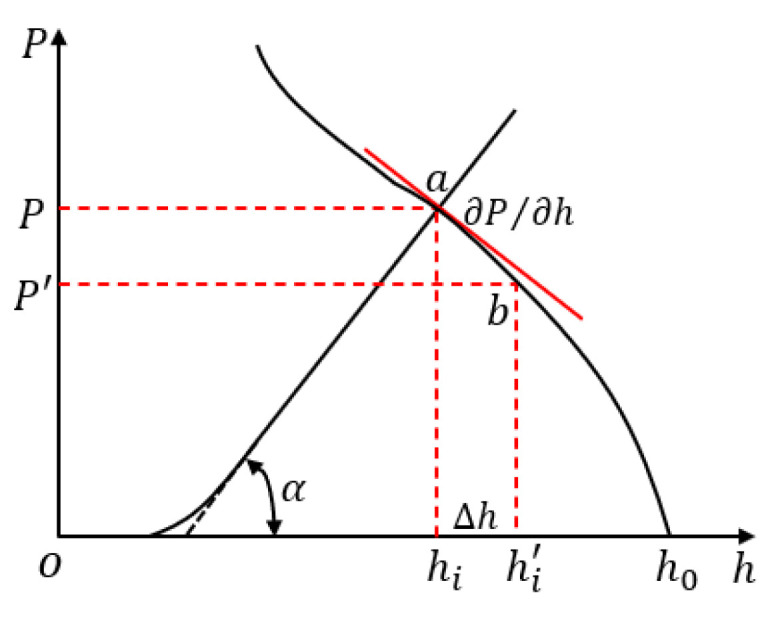
Schematic of secant method.

For the convenience of calculation, the secant method is used to calculate the partial score of the influence coefficient [34]. As shown in Figure 2, take the plastic deformation curve of the plate and strip during rolling as an example. In the rolling process, $∆h={0.001h}_{i}$ is the thickness fluctuation; thus, the new rolling force P can be obtained. Therefore, the partial differential coefficient can be expressed by the following formula.

(29) $$\frac{\partial P}{\partial h}=\frac{P-P^{\prime }}{h_{i}-h_{i}^{\prime }}$$

**Figure 3:**
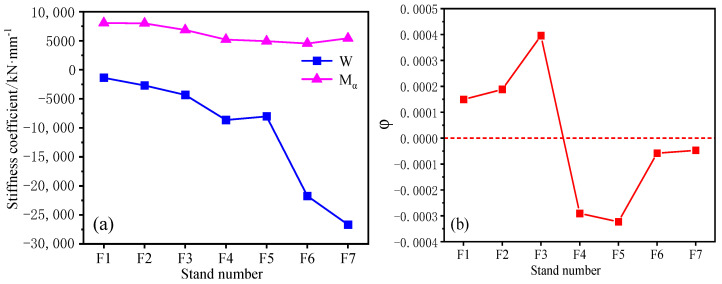
(**a**) Stiffness coefficient of each stand and (**b**) φ value.

The plastic stiffness coefficient and longitudinal stiffness modulus of the rolling mill of each stand is shown in Figure 3a. This paper selects the equivalent longitudinal stiffness coefficient of the rolling mill. Since the longitudinal stiffness modulus of the rolling mill and the plastic stiffness of the rolled parts are different in each stand, the φ parameter in the influence coefficient of the thickness difference of the strip behaves differently in each stand, as shown in Figure 3b. In the influence coefficient of thickness difference, the φ value is positive in F1–F3 stands and negative in F4–F7 stands. At the same time, the plastic stiffness coefficient of the rolled piece increases with the increase in rolling passes, and the difficulty of metal deformation increases.

Based on the incremental model of comprehensive static analysis, the influence coefficient of strip thickness difference in the steady rolling process can be calculated by the secant method. Based on the 1580 hot rolling historical data, the partial differential of each pass variable of 3.00 mm gauge strip with final rolling thickness was established. As shown in Table 2, partial differential values of rolling force, roll gap, and roll bending force need not be calculated.

In this paper, the partial score of the thickness difference influence coefficient was calculated by the secant method to calculate the thickness difference influence coefficient of the strip. Based on the influence coefficient of thickness difference, the influence weight of thickness difference of the strip based on the rolling mechanism is constructed. 

Based on the above static comprehensive analysis of the strip rolling process, the influence coefficients of each variable are calculated $\xi_{i}$ (The influence coefficient in Table 1 is expressed by $\xi_{i}$, i=1,2,⋯,m). Thus, in the rolling process, the influence of various influence factors on the longitudinal thickness difference can be seen through the influence coefficient $\xi_{i}$. Therefore, the influence coefficients of the above variables form the influence coefficient matrix ψ.

(30) $$\psi =\left(\xi_{1},\xi_{2},\cdots ,\xi_{m}\right)\in R^{m}$$

Mean value of each variable.

(31) $$\bar{X}=({\bar{X}}_{1},{\bar{X}}_{2},\cdots ,{\bar{X}}_{m})\in R^{m}$$

From above.

(32) $$I_{mm}=\psi^{T}\cdot \bar{X}=\left[\begin{array}{cc} \begin{array}{cc} I_{11} & I_{12} \end{array} & \begin{array}{cc} \cdots & I_{1m} \end{array}\\ \begin{array}{cc} \begin{array}{c} I_{21}\\ \vdots \\ I_{m1} \end{array} & \begin{array}{c} I_{22}\\ \vdots \\ I_{m2} \end{array} \end{array} & \begin{array}{cc} \begin{array}{c} \cdots \\ \ddots \\ \cdots \end{array} & \begin{array}{c} I_{2m}\\ \vdots \\ I_{mm} \end{array} \end{array} \end{array}\right]$$

Take the diagonal element. It is the influence weight of each variable that affects the abnormal fluctuation of the thickness of the strip in the hot rolling finishing mills.

(33) $$W=diag\left(I_{mm}\right)=\left(I_{11},I_{22},\cdots ,I_{mm}\right)$$

The normalized data set $\tilde{X}=[{\tilde{X}}_{1}^{1},{\tilde{X}}_{2}^{2},\cdots ,{\tilde{X}}_{m}^{n}]$ is weighted. Noted that $\tilde{X}$ is calculated in detail as shown in Appendix B. The data set is weighted and reconstructed. Reconstruct the data matrix as follows.

(34) $$X^{w}=\tilde{X}W=\left[\begin{array}{ccc} \begin{array}{c} {\tilde{x}}_{11}I_{11}\\ {\tilde{x}}_{21}I_{11} \end{array} & \begin{array}{c} \begin{array}{cc} {\tilde{x}}_{12}I_{22} & \cdots \end{array}\\ \begin{array}{cc} {\tilde{x}}_{22}I_{22} & \cdots \end{array} \end{array} & \begin{array}{c} {\tilde{x}}_{1p}I_{mm}\\ {\tilde{x}}_{2p}I_{mm} \end{array}\\ \vdots & \begin{array}{cc} \vdots & \ddots \end{array} & \vdots \\ {\tilde{x}}_{n1}I_{11} & \begin{array}{cc} {\tilde{x}}_{n2}I_{22} & \cdots \end{array} & {\tilde{x}}_{np}I_{mm} \end{array}\right]$$

### 3.3. Monitoring and Diagnosis Model Based on Weighted Data Reconstruction

Through the analysis of the influence weight of thickness quality, the normalized data is weighted and reconstructed. The reconstructed data is used to monitor and diagnose quality-related faults. The monitoring and diagnosis method of hot rolled strip quality-related process based on WKPLS method is shown in Algorithm 2.
**Algorithm 2:** The WKPLS-based fault detection approach*Off-line training*:Step 1Collect process variable data X and quality variable data Y.Step 2Normalization of process data $\tilde{X}$ by Equation (35).Step 3Quality influence weight by Equation (33)Step 4Reconstruct the feature data set $X^{w}$ by Equation (34). Step 5Run Algorithm 1 to calculate T and U.Step 6Given confidence limit α, calculate thresholds $T_{\mathrm{ucl}}^{2}\left(\alpha_{0}\right)$ and $SPE_{\mathrm{ucl}}^{2}\left(\alpha_{0}\right)$ by Equation (15).*On-line detecting*:Step 1Collect on-line samples $x_{new}$. Additionally, the data reconstruction is carried out in Steps 2 to 4 of the offline training part.Step 2Calculate ${\bar{K}}_{new,i}^{\prime }$ by Equation (17).Step 3Calculate statistics $T^{2}$ and SPE by Equation (14).Step 4Calculate the contribution plot to identify the abnormal variables.

Based on the above quality-related fault-detection process, decisions can be made according to the following diagnosis logic.

$\left\{\begin{array}{c} T^{2}<T_{\mathrm{ucl}}^{2}\\ SPE<SPE_{\mathrm{ucl}}^{2} \end{array}\right.$, at this point, the quality is stable and there is no fault.

$\left\{\begin{array}{c} T^{2}>T_{\mathrm{ucl}}^{2}\\ SPE>SPE_{\mathrm{ucl}}^{2}\;\mathrm{or}\;SPE<SPE_{\mathrm{ucl}}^{2} \end{array}\right.$, at this point, the quality-related fault has occurred.

$\left\{\begin{array}{c} T^{2}<T_{\mathrm{ucl}}^{2}\\ SPE>SPE_{\mathrm{ucl}}^{2} \end{array}\right.$, at this point, the quality-unrelated fault has occurred.

**Figure 4:**
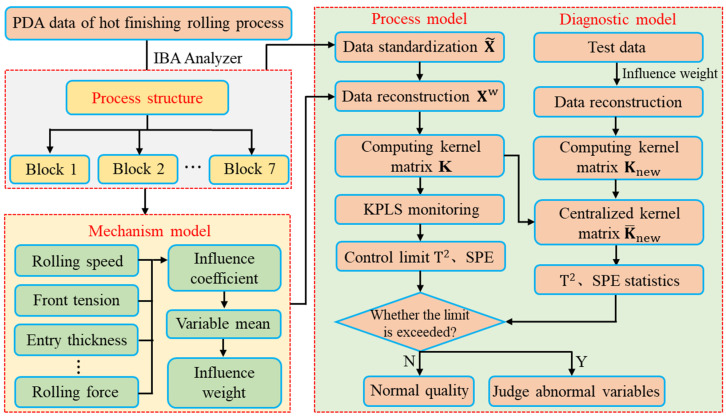
KPLS monitoring and diagnosis model based on weighted data reconstruction.

The detailed steps of the fault detection method based on WKPLS are summarized as Algorithm 2. The model framework is shown in Figure 4.

## 4. Experiments and Results Analysis

### 4.1. Data Preprocessing

To verify the feasibility of the proposed methods for abnormal diagnosis and path identification of quality-related variables, 1580 mm hot finishing production data were collected from the process data acquisition (PDA) system, and the required data were read by IBA analyzer. The hot rolling line mainly produces stainless steel, containers, high-end steel, and other products. Its product specification is steel plate and strips with a thickness of 1.2 mm~12.7 mm and width of 800 mm~1400 mm. In the rolling process, the rolling speed can reach more than 20 m/s, so the sampling interval of the actual production process data is 20–50 ms per point. Therefore, the data of strips with the same specifications (same specification, same slab thickness, same target thickness) are selected from the historical database to verify the model. In this paper, the production data of Q345B are used for industrial verification. The main variables include 43 process variables and 1 quality variable (Thickness), as shown in Table 3.

### 4.2. Influence Weight of Each Stand Variable

To improve and optimize the data model, the model is verified by the above hot rolling process data. Table 4 shows each stand variable’s influence coefficient on the strip’s thickness. Due to the order of magnitude of each variable, it is difficult to directly use this influence coefficient for the optimization of the data model. Therefore, considering that the influence of dimensionality is eliminated after data standardization, this paper uses the mean value of each variable to optimize the influence coefficient. The average value of each stand variable is shown in Table 5. 

**Figure 5:**
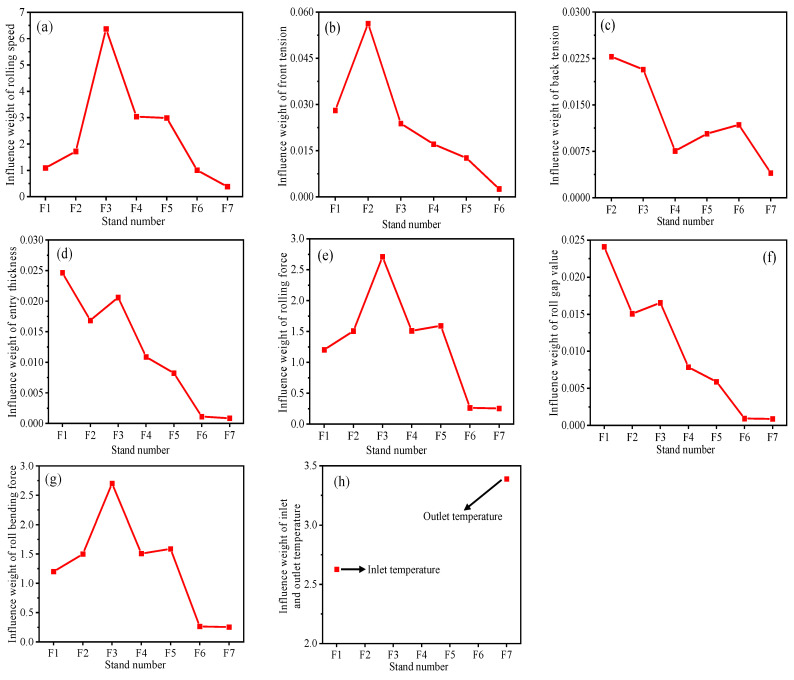
Influence weight of each stand variable. (**a**) Influence weight of rolling speed. (**b**) Influence weight of front tension. (**c**) Influence weight of post-tension. (**d**) Influence weight of entry thickness. (**e**) Influence weight of rolling force. (**f**) Influence weight of roll gap value. (**g**) Influence weight of roll bending force. (**h**) Influence weight of inlet and outlet temperature.

For the 1580 mm hot rolling process, the influence weight of each stand variable on the thickness is calculated. The weight of each variable’s impact on quality is shown in Figure 5. Figure 5a shows the influence weight of rolling speed, in which the influence weight of the F3 stand is significantly higher than that of other stands. With the increase of rolling passes, the degree of metal deformation decreases, and the influence weight shows a downward trend in the rear stand. Figure 5b,c show the influence weight of the front, and rear tension, in which the F7 stand has no front tension and the F1 stand has no post-tension. It can be seen that the influence of each stand’s front and rear tension on the thickness of the strip is different, and the influence weight of the front and rear tension of the F2 stand is the maximum. Figure 5d,f show the influence weights of the inlet thickness and the roll gap value, respectively. The inlet thickness causes the thickness deviation by affecting the rolling force and the roll gap value. The thickness difference of the strip is gradually corrected at each stand with the rolling passes, so the influence weight of the inlet thickness and the roll gap value shows a downward trend. Figure 5e,g show the influence weights of rolling force and bending force, respectively. From the analysis of the influence mechanism of strip thickness, it can be seen that the rolling force is the passive momentum, and the changes of various variables affect the rolling force, resulting in the thickness fluctuation of the strip, and the bending roll is an important control means of strip crown and shape in the rolling process. Therefore, the influence weight of rolling force and bending force on thickness shows a low value. Figure 5h shows the influence weights of the inlet and outlet temperatures of finishing rolling, and the outlet temperature of finishing rolling has a greater influence on the thickness.

Based on the above introduction, in the finishing rolling stage, the influence of each variable on the thickness is different. The first four passes greatly influence the thickness of the strip and play a leading role in the thickness control. The last three passes are mainly controlled by shape; thus, the influence on the thickness is reduced.

### 4.3. Thickness Monitoring Results Based on PLS and KPLS

**Figure 6:**
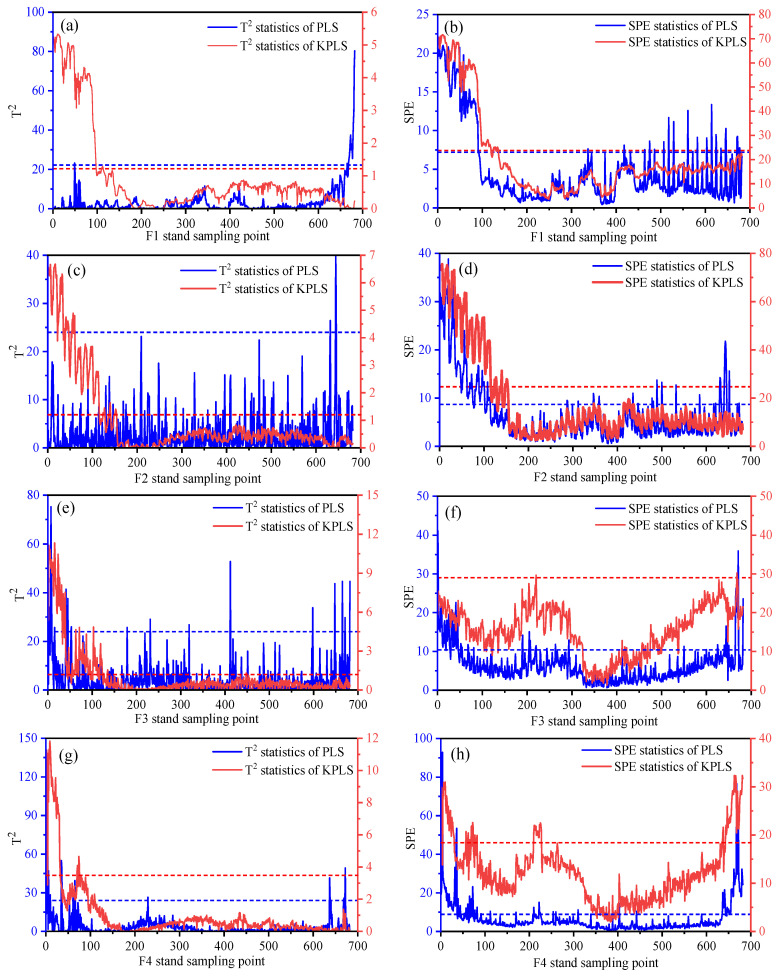

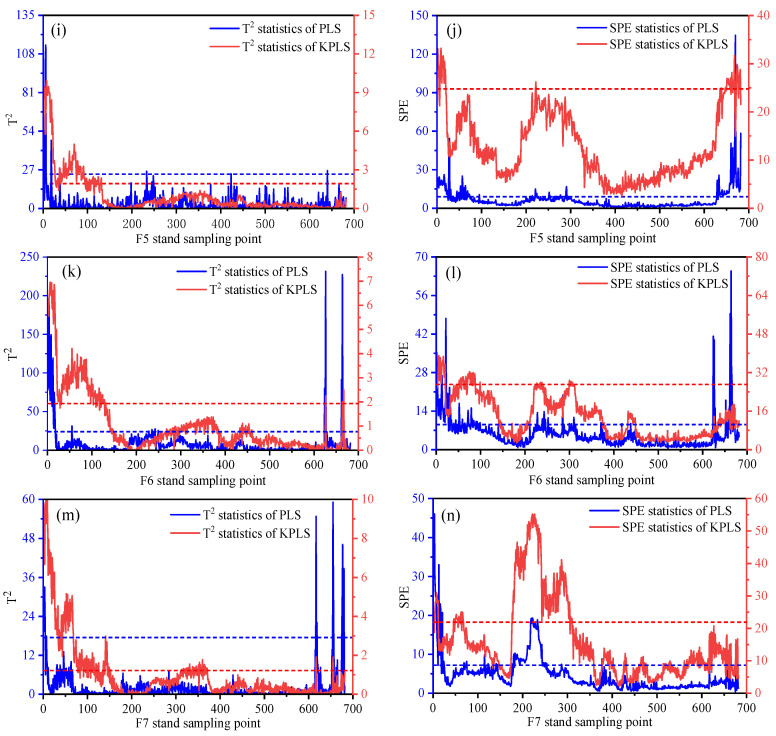
Monitoring statistics of each stand in the finishing rolling stage based on PLS and KPLS. (**a**) F1 stand $T^{2}$ statistics. (**b**) F1 stand SPE statistics. (**c**) F2 stand $T^{2}$ statistics. (**d**) F2 stand SPE statistics. (**e**) F3 stand $T^{2}$ statistics. (**f**) F3 stand SPE statistics. (**g**) F4 stand $T^{2}$ statistics. (**h**) F4 stand SPE statistics. (**i**) F5 stand $T^{2}$ statistics. (**j**) F5 stand SPE statistics. (**k**) F6 stand $T^{2}$ statistics. (**l**) F6 stand SPE statistics. (**m**) F7 stand $T^{2}$ statistics. (**n**) F7 stand SPE statistics.

**Figure 7:**
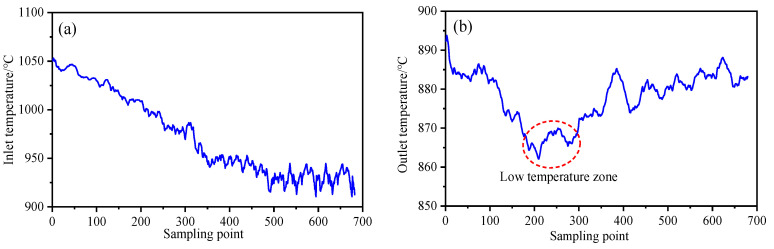
Hot rolled strip inlet and outlet temperature. (**a**) Finish rolling inlet temperature. (**b**) Finish rolling outlet temperature.

In the feature space, Hotelling’s $T^{2}$ and SPE statistics are used to calculate the statistical monitoring indicators, and the statistical control limits of $T^{2}$ and SPE are determined through the training data. The monitoring results of variables related to strip thickness and quality through PLS and KPLS monitoring methods are shown in Figure 6. Due to the nonlinearity between multivariate variables, as shown in Figure 6a–f, a large number of sample points exceeded the limit in the PLS monitoring process of F1–F3 stand, resulting in a large number of misjudgments. The monitoring statistics show a narrowing trend, showing more accurate monitoring results for the fluctuations of variables related to the quality of the production process. The thickness fluctuation of the head and tail of the strip is caused by the lack of complete tension control of the strip in the biting stage at the initial stage of rolling and the loss of tension at the tail of the strip at the end of rolling. From the statistical monitoring process in Figure 6g–n, it can be seen that the 0–100 sample points at the head and 650–680 sample points at the tail of the strip steel exceed the control limit. In the stable rolling state, the sample points are within the control limit, and the product thickness quality is stable. However, in Figure 6g–n, the statistical monitoring process of SPE on the residual subspace showed significant fluctuations at 200–300 sample points but did not cause quality abnormalities. It shows that some process variables may fluctuate greatly in the rolling production process. The inlet and outlet temperatures of the strips extracted from the production data of this batch of strips are shown in Figure 7. Figure 7b shows that there is a temperature depression at the outlet temperature of 200–300 sample points in the rolling stage, which is consistent with the fluctuation stage of SPE statistical monitoring. The uneven distribution of outlet temperature is the main reason for the large thickness fluctuation in the strip rolling process. The $T^{2}$ statistic of each stand does not exceed the limit after 100 sample points, and the system operates normally and is in a controlled state.

### 4.4. Thickness Monitoring Results Based on Mechanism Data Fusion

**Figure 8:**
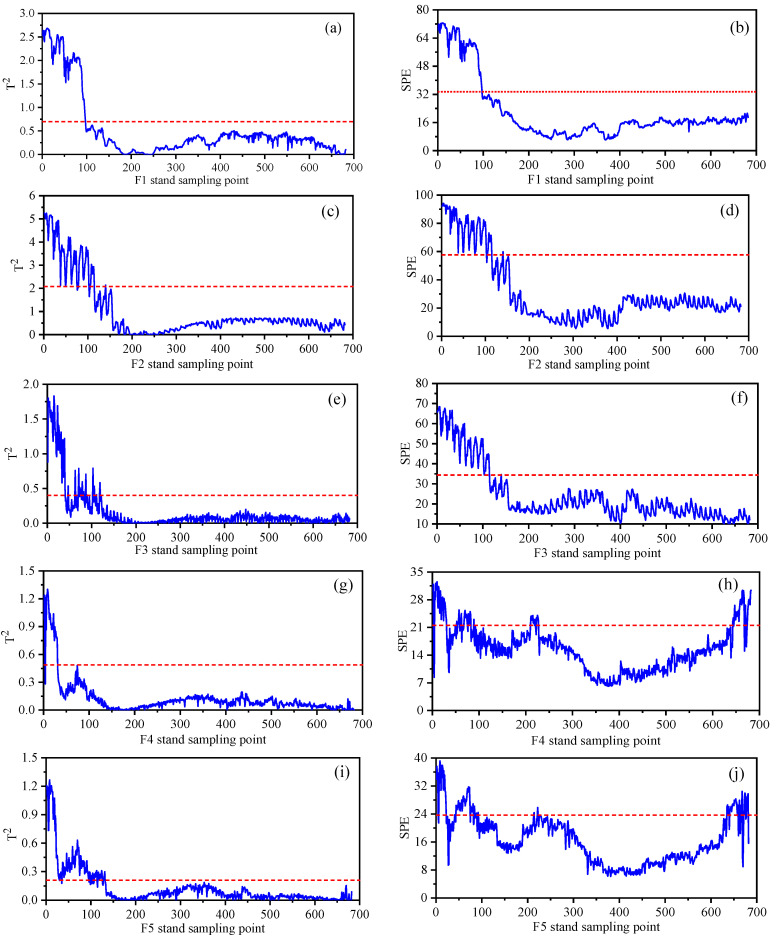

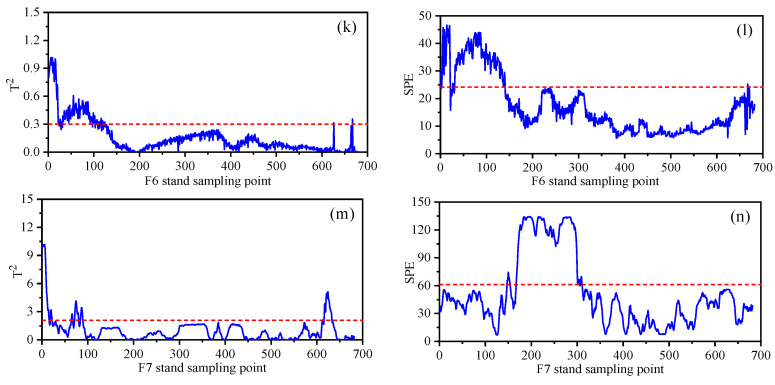
Monitoring statistics of each stand in the finishing rolling stage based on mechanism and data fusion. (**a**) F1 stand $T^{2}$ statistics. (**b**) F1 stand SPE statistics. (**c**) F2 stand $T^{2}$ statistics. (**d**) F2 stand SPE statistics. (**e**) F3 stand $T^{2}$ statistics. (**f**) F3 stand SPE statistics. (**g**) F4 stand $T^{2}$ statistics. (**h**) F4 stand SPE statistics. (**i**) F5 stand $T^{2}$ statistics. (**j**) F5 stand SPE statistics. (**k**) F6 stand $T^{2}$ statistics. (**l**) F6 stand SPE statistics. (**m**) F7 stand $T^{2}$ statistics. (**n**) F7 stand SPE statistics.

The above statistical process of quality-related process monitoring based on the data method shows the fluctuation of variables related to strip quality. To analyze the causes of abnormal fluctuation of strip quality, we build a contribution plot to identify variables. When constructing the contribution plot based on the traditional monitoring method, it is difficult to realize the variable root cause diagnosis when multiple variables’ contribution value is large because each stand’s variables in the rolling process have different influences on the quality. Therefore, based on the mechanism model, the influence weight of quality-related variables is constructed, and the strip quality-related process monitoring and diagnosis model based on the integration of the mechanism and data model are established. The monitoring model can achieve higher fault detection accuracy through the mechanism model to explain the input variables of the data model. Figure 8 shows the monitoring statistics of abnormal plate thickness based on the fusion of the rolling mechanism and data algorithm.

Comparing the monitoring statistics in Figure 6 and Figure 8, it can be seen that the statistical monitoring process of the fusion mechanism is highly consistent with the monitoring statistics based on the KPLS algorithm. It can be seen from the $T^{2}$ statistical process that the coordinate value of the $T^{2}$ statistic of the fusion mechanism is reduced because the monitoring process integrates the weight of the rolling mechanism and reconstructs and optimizes the strong correlation and weak correlation variables of quality. According to the SPE monitoring statistics of F3 in Figure 6f and Figure 8f, the fusion mechanism weight monitoring process is more stable. In the process of monitoring 0–130 sample points of the F6 stand, the mechanism data fusion monitoring shows a higher recognition rate, as shown in Figure 6l and Figure 8l. In Figure 6n and Figure 8n, although SPE statistics can identify 190–300 sample points, the latter shows a more stable monitoring state. Therefore, the quality-related fault monitoring model based on mechanism data fusion has better stability. This method is more obvious for the expression of data features.

### 4.5. Cause Identification of Quality Abnormity Based on Contribution Plot

**Figure 9:**
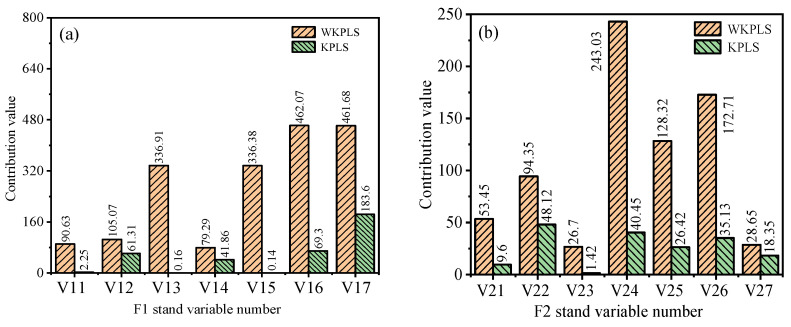

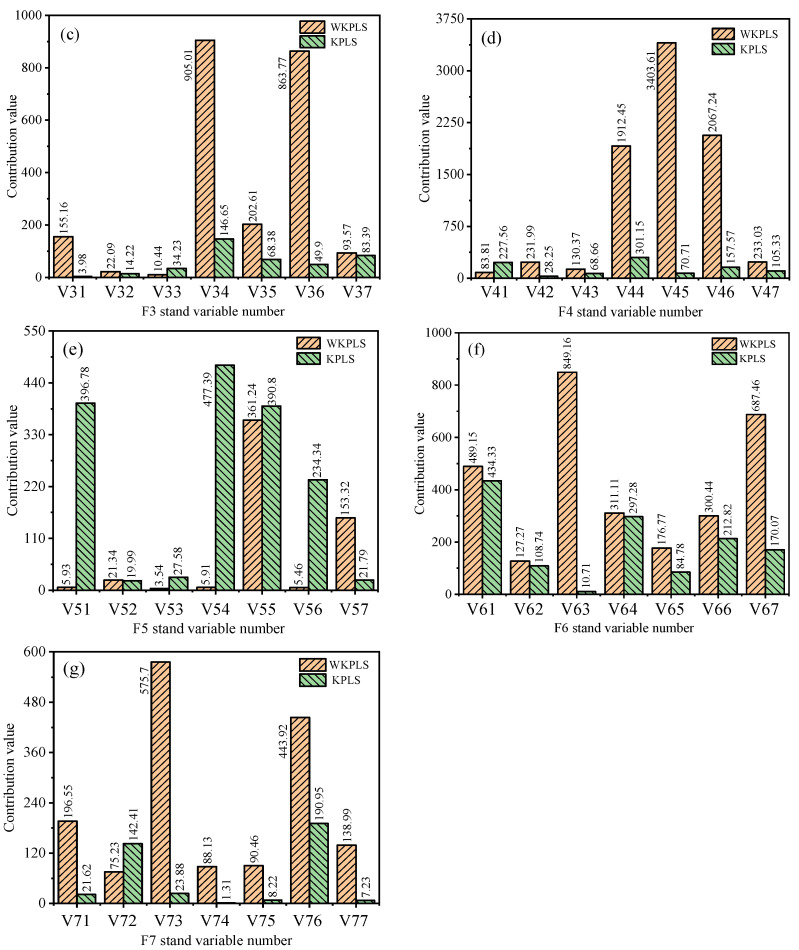
Contribution plot of each variable of the stand. (**a**) F1 stand variable contribution plot. (**b**) F2 stand variable contribution plot. (**c**) F3 stand variable contribution plot. (**d**) F4 stand variable contribution plot. (**e**) F5 stand variable contribution plot. (**f**) F6 stand variable contribution plot. (**g**) F7 stand variable contribution plot.

To identify the causes of quality defects, the contribution map of each variable to quality-related anomalies is constructed using the contribution plot, as shown in Figure 9.

Combined with the statistical monitoring results, 1–100 sample points are out of tolerance, and 190–300 sample points gradually fluctuate sharply in the SPE statistics of F3–F7 stands. This sample point exceeds the control limit in Figure 8n. From the contribution plot based on the KPLS algorithm in Figure 9, we can see the contribution value of each stand variable to the strip quality. However, this method is difficult to effectively distinguish the main abnormal variable from the secondary fault variable when there are large anomalies in multiple variables in the process of fault variable identification. As shown in Figure 9b–d, the contribution value of unmerged mechanism weight and the contribution values of each variable are not different, which is easy to cause misjudgments and the cause of abnormal quality in the diagnosis process. Therefore, this paper considers the influence mechanism of strip thickness in the rolling process and establishes the influence weight of thickness based on the influence mechanism. The monitoring and diagnosis model of strip quality-related faults is established based on mechanism data fusion. Effectively improve the recognition of the contribution value of variables related to the quality of the rolling process. 

At the entrance of the finishing mill, the temperature of the strip head drops due to the use of high-pressure water for descaling, which makes the overall temperature of the slab uneven and causes the thickness deviation of the strip head. Through the diagnosis of the method in this paper, it can be determined that the inlet temperature of the F1 stand is the main reason for the head thickness deviation, which is consistent with the field test results, as shown in Figure 9a. At the same time, the contribution value of each variable of the F1 stand fluctuates to varying degrees. Because the head thickness is out of tolerance, the contribution value of F2 stand Var24 (Entry thickness) is the largest, which is the main reason for the monitoring statistics of this stand to exceed the limit. According to the analysis of the rolling mechanism, the change of inlet thickness causes the fluctuation of Var25 (Rolling force) and Var26 (Roll gap value), which become the secondary fault source of the diagnosis of the stand, as shown in Figure 9b. Due to the quality transfer characteristics between processes, the thickness difference of the strip head in the last pass will lead to fluctuations in the variable inlet thickness, rolling force, and roll gap values in the subsequent rolling process, as shown in Figure 9c–e. With the increase in rolling passes, F5-F7 stands are biased towards shape control, and the weight of each variable on strip thickness gradually decreases. Since the plate thickness and shape are coupled in the hot rolling process, Var63 (front tension) of the F6 stand and Var73 (post tension) of the F7 stand show the largest contribution to the longitudinal thickness difference of the strip, as shown in Figure 9f,g. Based on the influence mechanism of strip thickness in the rolling process, the change of tension affects the stress state of rolled pieces, which leads to the fluctuation of metal deformation resistance, and finally leads to the fluctuation of longitudinal thickness difference of strip. At the same time, the metal deformation resistance is affected by the change in rolling temperature. Extract the outlet temperature of the hot rolling 1580 production line. As shown in Figure 7b, there are temperature pits at 200–300 sample points, which makes the 200–300 sample points in the SPE monitoring statistical process in Figure 8n exceeds the limit. Therefore, the monitoring and diagnosis results of the variables related to the longitudinal thickness of the strip through the mechanism and data fusion are consistent with the actual production state. This paper establishes the thickness influence weight through the mechanism model. It integrates the data model, which can not only show the stability of the quality-related fault diagnosis process but also have a better detection rate in the monitoring and diagnosis of quality-related abnormal variables in complex industrial processes than in traditional algorithms, as shown in Table 6.

Therefore, the quality-related process monitoring and diagnosis method of hot-rolled strips based on the weighted feature KPLS proposed in this paper can better realize the diagnosis of quality-related strip faults. The new method has a better detection rate than the traditional algorithm in the monitoring and diagnosis of quality-related abnormal variables in complex industrial processes, and the average accuracy rate can reach 96%.

### 4.6. Fault Detection Rate and False Alarm Rate of WKPLS Diagnosis Method

However, although the diagnostic rate of quality abnormality reached 96%, there is still about 5.58% false alarm rate, as shown in Table 7. It can be seen from the literature [25,35] that the quality diagnosis method proposed in this paper does not have a better reduction in the rate of misjudgment. However, the proposed method strengthens the interpretation of process variables to quality variables, and improves the clarity of the diagnosis of causes affecting quality defects.

## 5. Conclusions

We aimed to solve the problem of traditional data models neglecting the physical significance of variables and the contribution of each variable to quality in the process of quality-related fault diagnosis. The above issues caused incorrect identification of abnormal variables during the diagnostic process. Therefore, a method for quality-related process monitoring and diagnosis of hot-rolled strips based on the weighted statistical feature KPLS was proposed. Through the analysis of industrial test results of the 1580 production line, the following conclusions were drawn.

(1) In this paper, the Gaussian radial basis kernel function is introduced to transform the nonlinear relationship into a linear relationship in the high-dimensional feature space. The ability of the model to process nonlinear data is improved.
(2) Based on the rolling mechanism model, the thickness influence weight is constructed. The input features of the data model are weighted by the influence weight, which improves the physical interpretation ability of the data model to each variable.
(3) The contribution diagram is introduced to identify the causes of abnormal thickness quality and determine the relevant variables that cause quality abnormalities.
(4) The industrial verification was carried out by using the measured data of 1580 production line. It can be seen that the monitoring and diagnosis model of strip thickness quality related fault combined with rolling mechanism has high detection accuracy, and the accuracy can reach 96%.

## Figures and Tables

**Table 1:** Influence coefficient of some variables on longitudinal thickness difference.

Name	Influence Coefficient	Expression	Name	Influence Coefficient	Expression
Roll gap influence coefficient	$Q_{S_{0}}^{S}$	$M_{\alpha }\cdot \varphi$	Front tension influence coefficient	$Q_{T_{f}}^{S}$	$\frac{\partial P}{\partial T_{f}}\cdot \varphi$
Influence coefficient of incoming material thickness	$Q_{H}^{S}$	$\frac{\partial P}{\partial H}\cdot \varphi$	Friction coefficient influence coefficient	$Q_{\mu }^{S}$	$\frac{\partial P}{\partial \mu }\cdot \varphi$
Influence coefficient of back tension	$Q_{T_{b}}^{S}$	$\frac{\partial P}{\partial T_{b}}\cdot \varphi$	Influence coefficient of deformation resistance of a rolled piece	$Q_{K}^{S}$	$\frac{\partial P}{\partial K}\cdot \varphi$

**Table 2:** Partial scores of variables of 1580 mm hot rolling stands.

Stand No.	$H_{0}$/mm	h/mm	The Partial Differential Value of Thickness Difference Influences Coefficient				
			Rolling Speed	Front Tension	Back Tension	Entry Gage	Temperature
F1	38.00	20.10	6069.20	28.86	-	8496.15	17.92
F2	20.10	11.90	4340.37	33.74	18.58	8691.76	-
F3	11.9	7.15	4557.55	5.17	5.89	9293.33	-
F4	7.15	5.30	2004.74	4.28	2.22	7814.92	-
F5	5.30	3.79	1345.66	2.30	2.34	6893.20	-
F6	3.79	3.35	2082.96	2.25	11.95	5500.63	-
F7	3.35	3.00	864.60	-	4.24	5168.80	81.92

**Table 3:** Names of variables in the finishing rolling stage of hot-rolled strip.

Variable Name	F1	F2	F3	F4	F5	F6	F7
Rolling speed (m/s)	V11	V21	V31	V41	V51	V61	V71
Front tension (MPa)	V12	V22	V32	V42	V52	V62	-
Post tension (MPa)	-	V23	V33	V43	V53	V63	V72
Entry thickness (m)	V13	V24	V34	V44	V54	V64	V73
Rolling force (MN)	V14	V25	V35	V45	V55	V65	V74
Roll gap value (m)	V15	V26	V36	V46	V56	V66	V75
Roll bending force (N)	V16	V27	V37	V47	V57	V67	V76
Inlet temperature (°C)	V17	-	-	-	-	-	-
Outlet temperature (°C)	-	-	-	-	-	-	V77
Thickness (m)	-	-	-	-	-	-	h

**Table 4:** Variable influence coefficient of 1580 mm hot rolling stands.

Stand No.	Influence Coefficient							
	Rolling Speed	Front Tension	Post Tension	Entry Thickness	Rolling Force	Roll Gap Value	Roll Bending Force	Temperature
F1	0.90721	0.00431	-	1.270	0.0000499	1.20478	0.00064	0.00268
F2	0.81740	0.00635	0.00350	1.637	0.0000606	1.50659	0.00057	-
F3	1.80425	0.00205	0.00233	3.679	0.0001224	2.71180	0.00118	-
F4	0.58210	0.00124	0.00065	2.269	0.0001051	1.50987	0.00127	-
F5	0.43436	0.00074	0.00075	2.225	0.0001390	1.59135	0.00199	-
F6	0.12107	0.00013	0.00069	0.320	0.0000286	0.26331	0.00099	-
F7	0.04069	-	0.00020	0.243	0.0000336	0.25505	0.00215	0.00385

**Table 5:** Mean value of variables of 1580 mm hot rolling stands.

Stand No.	Mean Value of Each Variable							
	Rolling Speed (m/s)	Front Tension (MN)	Post Tension (MN)	Entry Thickness (m)	Rolling Force (kN)	Roll Gap Value (m)	Roll Bending Force (kN)	Temperature (°C)
F1	1.20	6.51	-	0.0194	24,130	0.02	1877.27	980
F2	2.10	8.86	6.51	0.0103	24,870	0.01	2629.76	-
F3	3.53	11.61	8.89	0.0056	22,130	0.0061	2290.81	-
F4	5.22	13.79	11.61	0.0048	14,370	0.0052	1187.13	-
F5	6.88	17.06	13.77	0.0037	11,450	0.0037	798.03	-
F6	8.30	19.91	17.05	0.0035	9190	0.0035	266.12	-
F7	9.32	-	19.95	0.0035	7600	0.0034	118.78	880

**Table 6:** Abnormal detection rate of relevant variables of strip quality in the rolling process.

Method	PLS	KPLS	TPLS	WKPLS
Detection rate	0.66	0.87	0.9	0.96

**Table 7:** Fault detection rate and false alarm rate of WKPLS diagnosis method.

Steel Coil Number	Specifications (mm)	Fault Detection Rate (%)	False Alarm Rate (%)
8E00367A20xxAxxx	3 × 870	97.21	4.12
8E00323A30xxAxxx	3 × 866	95.60	6.42
8E00324A10xxAxxx	3 × 866	94.14	6.61
8E00327A20xxAxxx	3 × 861	98.02	4.33
8E00328A50xxAxxx	3 × 866	95.60	6.42
Mean		96.11	5.58

## Data Availability

The experimental data can be obtained on request from guohesong@stumail.ysu.edu.cn (H.G.). The experimental data is obtained through the National Engineering Research Center for Equipment and Technology of Cold Rolled Strip of Yanshan University. The experimental results are reproducible.

## Appendix A

According to the bouncing equation, the increment of thickness is taken as follows.

(A1) $$∆h=∆S_{0}+\frac{∆P}{M}-∆\delta$$

The above formula includes the increment of no-load roll gap $∆S_{0}$, the increment of rolling pressure ∆P, the increment of oil film thickness of roll bearing ∆δ and the longitudinal stiffness modulus M of the rolling mill.

Under the condition that the width of rolled piece and the radius of roll are fixed, the rolling pressure is actually a function of the thickness of the rolled piece, tension, friction coefficient, deformation resistance of rolled piece and other factors [36], which can be expressed as.

(A2) $$P=P\left(H,h,T_{f},T_{b},\mu ,K\right)$$

where, H and h are the thickness before and after rolling, $T_{f}$ is the front tension, $T_{b}$ is the post-tension, μ is the friction coefficient, and K is the deformation resistance of the rolled piece, respectively.

By expanding the rolling pressure Equation (A2) according to the Taylor series and ignoring the higher order, the higher order equation of the rolling pressure can be obtained:

(A3) $$∆P=\frac{\partial P}{\partial h}\cdot ∆h+\frac{\partial P}{\partial H}\cdot ∆H+\frac{\partial P}{\partial T_{b}}\cdot ∆T_{b}+\frac{\partial P}{\partial T_{f}}\cdot ∆T_{f}+\frac{\partial P}{\partial \mu }\cdot ∆\mu +\frac{\partial P}{\partial K}\cdot ∆K$$

Formula (A3) can be written as follows:

(A4) ∆P=−W·∆h+∆F

where, W is the plastic stiffness coefficient of the rolled piece, W=−∂P/∂h. ∆F is the change of rolling force caused by external interference ∆H, $∆T_{b}$, $∆T_{f}$, ∆μ, ∆K, which is expressed by the following formula.

(A5) $$∆F=\frac{\partial P}{\partial H}\cdot ∆H+\frac{\partial P}{\partial T_{b}}\cdot ∆T_{b}+\frac{\partial P}{\partial T_{f}}\cdot ∆T_{f}+\frac{\partial P}{\partial \mu }\cdot ∆\mu +\frac{\partial P}{\partial K}\cdot ∆K$$

Combine formula (A1) and formula (A4) to obtain.

(A6) $$∆h=\left(∆S_{0}-∆\delta +\frac{∆F}{M}\right)/\left(1+\frac{W}{M}\right)$$

Since rolling bearing is used in 1580 mm hot rolling production line, ∆δ=0.

## Appendix B

Due to the large difference in data distribution and range between production process variables, the mean ${\bar{x}}_{j}$ and standard deviation $s_{j}$ are used to normalize each variable to eliminate the impact of dimensions. The normalization method used in this paper is shown in the following formula.

(A7) $${\tilde{x}}_{ij}=\frac{x_{ij}-{\bar{x}}_{j}}{s_{j}}$$

Composition vector.

(A8) $${\tilde{X}}_{j}^{i}={\left[{\tilde{x}}_{11},{\tilde{x}}_{21},\cdots ,{\tilde{x}}_{n1}\right]}^{T}\left(i=1,\cdots ,n;j=1,\cdots ,m\right)$$

The normalized data set.

(A9) $$\tilde{X}=\left[{\tilde{X}}_{1}^{1},{\tilde{X}}_{2}^{2},\cdots ,{\tilde{X}}_{m}^{n}\right]$$
